# Lifestyle and health behaviour change support in traditional acupuncture: a mixed method survey study of reported practice (UK)

**DOI:** 10.1186/s12906-022-03719-6

**Published:** 2022-09-21

**Authors:** J. Pinto, K. Bradbury, D. Newell, F. L. Bishop

**Affiliations:** 1 Department of Psychology, Faculty of Environmental & Life Sciences, University of Southampton, Highfield Campus, Southampton, SO17 1BJ UK; 2 Faculty of Medicine, University of Southampton, Highfield Campus, Southampton, SO17 1BJ UK; 3 AECC University College, Bournemouth, BH5 2DF UK

**Keywords:** Health behaviour, Lifestyle, Acupuncture, Behaviour change, Health Promotion, Self-care

## Abstract

**Aims:**

Complementary medicine therapists such as traditional acupuncturists are a large resource for supporting public health targets to improve health behaviours. Our objectives were to determine the prevalence and patterns of UK acupuncturists’ provision of lifestyle change support, test theory-based hypotheses about facilitators and barriers to supporting lifestyle changes and to explore associated characteristics and attitudes.

**Methods:**

A mixed methods design in which British Acupuncture Council members (Sept 2019-April 2020) completed an online questionnaire assessing prevalence of lifestyle change support, typical patterns across patients and behaviours, Theory of Planned Behaviour constructs, practitioner characteristics and open-text responses regarding additional behaviours and clinical decisions to introduce lifestyle change.

**Results:**

Three hundred fifty-two traditional acupuncturists participated (Mean age = 51.5 years, SD 9.9; 81.8% (*n* = 288) female). 57.7% (*n* = 203) reported offering support for lifestyle change during their most recent consultation. 91.7% (*n* = 323) reported supporting lifestyle change ‘always or most of the time’ for patients with chronic conditions and 67.9% (*n* = 239) reported this for patients with acute conditions. The pattern of typical support for different health behaviours ranged from 44.6% (*n* = 157) for smoking reduction (acute conditions) to 95.2% (*n* = 335) for diet support (chronic conditions). A linear regression model found that frequency of support for lifestyle change in acute patients was predicted by acupuncturists’ attitudes to both clinical role and importance of health behaviours, confidence in their ability to provide lifestyle change support and use of fewer behaviour change techniques. The decision to first offer lifestyle change support was guided by perceived patient receptiveness, whether presenting condition/diagnosis were likely to improve with lifestyle change and whether a strong therapeutic relationship was established.

**Conclusions:**

Traditional acupuncturists’ reports suggest their work supports key public health targets for promoting healthy behaviours. Less frequent support for alcohol/smoking may reflect user characteristics but may suggest training needs for acupuncturists. Increase could be made for support in acute presentations, however the importance of patient receptiveness, linking advice to condition, and therapeutic alliance should be explored further. There may be important differences between acupuncture practice and mainstream healthcare (e.g. high level of contact, longer visits, holistic approach) which impact mechanisms of action of behaviour change.

**Supplementary Information:**

The online version contains supplementary material available at 10.1186/s12906-022-03719-6.

## Background

Four key health behaviours: diet, physical activity, smoking and alcohol consumption, are now established as primary causes of ill health in the UK [1]. There is also growing evidence that poor sleep is a major cause of poor health outcomes [2]. Addressing behavioural causes of ill health is a public health imperative and possible approaches include expanding the public health workforce and better harnessing existing expertise.

A Royal Society for Public Health report [3] has set an agenda for a wider workforce to support core public health workers, particularly in promoting changes in modifiable health behaviours. This agenda includes understanding training needs, forming educational partnerships, developing an evidence base and evaluation tools for interventions.

There is an ‘untapped’ resource of Complementary Medicine (CM) providers for promoting health behaviour change [4, 5]. The Professional Standards Authority (PSA) Accredited Registers workforce represents 80,000 UK health care providers including acupuncturists, nutritional therapists, play therapists, sports rehabilitators, counsellors and psychotherapists and a variety of other complementary therapists. A survey conducted by the PSA in 2017 found that three quarters of registered practitioners feel that they are under-utilised in promoting the public’s health [5]. Identifying the potential roles and training needs for CM providers regarding health promotion and health behaviour change is a priority. CM practitioners, working outside the Allied Health Professions, often in private practice, are somewhat disconnected from mainstream and public health organisations as well as research institutions. Hence there remains significant gaps in knowledge and a need for research which aims to understand current CM practice and users with regards to health promotion, preventative medicine and health behaviour change.

CM providers may be particularly well suited to supporting health behaviour change. CM users have a high prevalence of chronic disease and modifiable behaviours; prevention of disease and health maintenance are key reasons for attending CM, and typical high levels of contact with repeated visits of long duration may facilitate lifestyle and health behaviour change (LHBC) [4, 6]. Large scale surveys show patients report making health behaviour changes as a result of treatment from CM providers [7, 8] and CM usage is associated with belief that health is a result of personal behaviour that may include lifestyle [9].

Traditional acupuncture has a historical connection to healthy lifestyle practises and is often practised alongside other traditional treatments including massage, herbal medicine, dietary therapy and exercises such as tai-chi. Traditional acupuncture practice is regarded as a complex intervention containing multiple components including, but not limited to, acupoint needling/stimulation [10]. In the UK an estimated 4 million acupuncture sessions are provided annually (based on data from 2009) with approximately two-thirds of this provision outside the National Health Service (NHS), and approximately two-thirds provided by practitioners trained in traditional Chinese/East Asian styles of acupuncture [11]. Traditional acupuncture is used for a variety of conditions, in particular there is a large body of evidence for the use of acupuncture to treat long-term pain, migraine, back pain and knee osteoarthritis. The British Acupuncture Council (BAcC) is the main professional body for traditional acupuncturists in the UK and is on the PSA accredited register, representing over 2500 members. Acupuncture users report seeking treatment for disease prevention [12] and acupuncture use is associated with healthy behaviours [13]. Acupuncture use has been associated with greater physical activity in patients with breast and gynaecological cancer during oncology treatment [14]. The directionality of these associations is not clear, and there may be bidirectional influences between healthy behaviours and acupuncture use: individuals with healthy lifestyles are attracted to acupuncture (and other CM therapies), and acupuncture use may trigger adoption of healthy behaviours.

Our Critical Interpretive Synthesis (CIS) [15] review of acupuncture and LHBC which included 79 articles found the reported prevalence of LHBC support in acupuncture consultations varied widely*.* Some of this diversity could be explained by acupuncture providers being more likely to support LHBC for patients with chronic conditions than acute conditions (the 13% outlier lowest prevalence figure was from a study which included a large proportion (47.5%) of acute respiratory presentations) [16]. Our review found some evidence that acupuncturists are more likely to support diet and physical activity change than alcohol or smoking. There is insufficient trial evidence for the effectiveness of traditional acupuncture care on LHBC: some studies suggested an effect [17–19] but one trial found no effect (compared to self-care leaflet) at 6 and 12 month follow-up [20]. A large number of qualitative studies report that providers and users of acupuncture support and make LHBC [15, 21, 22].

Psychological theory suggests factors that may influence the extent to which acupuncturists provide LHBC support. The Theory of Planned Behaviour (TPB) suggests that volitional behaviour (in this case, offering LHBC support) occurs when traditional acupuncturists *intend* to offer this support, and that these intentions are themselves determined by the combination of *attitudes* to the behaviour; *subjective norms* (perceived social pressures to perform the behaviour); and *perceived behavioural control* (PBC) (the individual’s perception of their ability to perform the behaviour) [23]. Evidence suggests that the TPB can predict 20–30% of variance in behaviour [24]. The attitudes, norms and PBC of acupuncturists regarding LHBC support may therefore be associated with reported levels of LHBC support.

## Methods

Our first aim was to establish the prevalence of UK traditional acupuncturists support for LHBC and the patterns of support across different behaviours (particularly diet, physical activity, alcohol consumption, smoking and sleep hygiene) and across different patients (those presenting with acute/chronic conditions). Our second aim was to test which TPB variables and other practitioner characteristics predict acupuncturists’ engagement in LHBC support. Lastly, we explored what factors might guide clinical decisions about when to first offer LHBC support.

### Design

A concurrent (embedded) mixed methods approach was used. Participants completed a questionnaire online on a single occasion. The emphasis was on quantitative data to address the primary aims (establishing prevalence, predictors and associations for LHBC support). A number of open-ended questions were asked in order to explore the range of different lifestyle areas addressed by acupuncturists and their reasons for decisions regarding offering LHBC, in order to achieve a more complete understanding. These qualitative data were integrated with quantitative results during analysis (using content analysis to create quantitative data) and interpretation.

### Development of questionnaire

The questionnaire was informed where possible from previous validated instruments and our CIS review [15]. Items were refined to make the questions appropriate for the scope of practice of traditional acupuncturists and to reflect our focus on the five key health behaviours. See downloaded questionnaire in Additional file 1: Appendix 1.

1. Prevalence of LHBC work. Based on the principle of taking a single snapshot in time from a sample of a defined population of traditional acupuncturists (BAcC members). Further questions asked about typical practice for acute (defined as symptoms < 3 month duration) and chronic (defined as symptoms > 3 months duration) conditions and different health behaviours.
2. Scale items measuring key theoretical constructs of TBP (attitudes, PBC and norms) were based on four surveys of health promotion and lifestyle counselling by other health professionals, with relevant modifications:

- Attitudes to Clinical Role – (finding out about patients’ health behaviours and responsibility for LHBC support)

Attitudinal items were based on previous questionnaire [25] language altered and some items removed to reflect the different scope of practice (e.g. amended ‘preventative medicine’ to ‘lifestyle issues’) and to shift the focus more broadly to LHBC work (e.g. changed ‘health promotion’ to ‘lifestyle issues’). We amended the items following the statement “Identification of the following risk factors is a very important part of my day-to-day work” to reflect the focus on the key five health behaviours already outlined. We removed two statements: “My job is not only to treat disease but act as a health educator” to keep the focus on support for change rather than health education and “Practice nurses are the most appropriate to carry out health promotion”.

Seven items with seven-point Likert scale (strongly agree to strongly disagree).

b) Attitudes to Behaviours (importance of health behaviours)

Based on two surveys of physicians and nurses [26, 27]. These surveys asked respondents to rate different lifestyle behaviours and preventative care services in terms of importance in promoting good health in patients. The behaviours were altered for this survey to reflect the scope of practice of traditional acupuncturists (i.e. removing medical preventative care services) and to reflect the key health behaviours (smoking, diet, alcohol, physical activity and sleep hygiene).

Five items with five-point Likert scale (very important to not at all important).

iii) Perceived Behavioural Control (PBC)

These items were based on previous questionnaires [25, 28] again language was altered to reflect LHBC support more broadly (e.g. “counselling advice” changed to “address lifestyle issues”). One item ‘I can offer my patients a great deal in the way of lifestyle counselling’ [25] was amended to ‘I am confident in my ability to help patients change their lifestyle habits’.

Three items with seven-point Likert scale (strongly agree to strongly disagree).

iv) Social Norms – (regarding clinical engagement in LHBC support)

Items based on previous questionnaire survey of health promotion capacity [28] with amendments to wording e.g. from “health promotion” to “promotion of lifestyle change” to reflect our focus and from “most professionals” to “traditional acupuncturists”.

Three items with seven point Likert scale (strongly agree to strongly disagree).

3) Reported use of specific Behaviour Change Techniques (BCTs). 20 BCTs selected from the BCT Taxonomy [29] which two researchers (one qualified acupuncture practitioner) judged to be most likely applicable.
4) Demographics/Characteristics. Items included: gender; age; years in practice; treatments provided p/week; clinical setting; and main acupuncture style (styles were taken from previous in-house BAcC membership survey with open text option for respondents to report in their own words).
5) Optional: Personal lifestyle habits. Based on the Simple Lifestyle Indicator Questionnaire [30].

### Cognitive interviews – fine tuning the questionnaire

Prior to commencing the survey, cognitive ‘think aloud’ interviews were undertaken to refine the questionnaire. The process involves a researcher sitting next to a participant, audio-recording and making notes as the participant works through a questionnaire saying aloud whatever comes to their mind [31]. Participants were BAcC members recruited through personal contacts of the researchers. Eight participants took part in interviews. Findings were used to refine the language in the questionnaire to improve relevance and understanding among respondents.

### Participants: sampling and data collection

All practicing members of the BAcC at the time of the survey were eligible to participate. The survey was distributed electronically via the online platform Qualtrics. BAcC members were recruited through the organisation’s eNews bulletins, magazine and annual conference and reminders were posted on social media. The survey was open from September 2019 to April 2020. Average completion time for the survey was 40 minutes. A £5 gift voucher incentive was offered to all participants.

A sample size calculation was performed to obtain a reliable figure for prevalence of providing LHBC support. Based on total population of practicing full BAcC members 2407, confidence level of 95% and error margin of 5% and resulting in a target sample size of 332.

### Statistical methods

#### Scale development

Likert scale questions across four domains: Attitudes to Behaviour, Attitude to Clinical Role, Norms, and Perceived Behavioural Control (PBC) were analysed using a Principal Component Analysis (PCA) and Cronbach’s Reliability test (See Additional file 2: Appendix 2).

#### Power calculation, missing data and imputations

A power calculation indicated that minimum sample size for multiple regression was 108, given alpha = 0.05, 8 predictors in the model, the anticipated effect size of 0.15, and power = 0.8. The original dataset had 439 respondents. Cases were removed if they were ineligible (*n* = 58) or only answered up to first two blocks of questions (*n* = 29). Three hundred fifty-two cases retained. The overall proportion of missing values was very low (0.426%). A single imputed data-set was used for the rest of the analysis [32] (results of multiple regression tests were compared to complete case analysis/original data and there was no discrepancy).

#### Regression analysis on predictors of typical support for LHBC

Regression analysis was performed to identify key facilitators and barriers to acupuncturists’ engagement with LHBC support for patients presenting with acute conditions. The data for typical practice in chronic conditions was not suitable for regression analysis (did not have normal distribution/had very low scores on the negative side of the scale - with only 11 participants selecting *5-Never* or *4-Sometimes* in response to the question ‘How frequently do you try to help these patients change their lifestyle?’). The data for typical practice with acute conditions was suitable for regression analysis (was a measure of numeric frequency with a meaningful mid-point, data was more normally distributed and there was no problem with normality of standardised residuals). After checking assumptions for the multiple regression analysis one variable (Eight Principles Style) with only 5 cases was removed, and three outlier cases were removed. There were no problems with multicollinearity (all VIF scores less than 1.5 and tolerance larger .60).

A simple univariate regression analysis was undertaken to establish any potential significant correlations. The significant predictors which met assumptions for multiple regression were then then entered into a multiple linear regression model using a hierarchical approach, entering the eight variables in separate blocks guided by previous theory and effect size as determined in the univariate analysis.

### Qualitative data analysis

Content analysis was undertaken on open-text data which provided information on ‘other’ lifestyle change issues in order to inductively produce a set of manifest themes to describe the main areas, and findings were integrated with quantitative findings. Qualitative data which provided information about practitioners’ reasons for deciding when to first offer LHBC support was also coded using content analysis with the additional step of quantifying codes for comparison with other quantitative data. A code book was created using an inductive process. Each coding unit (defined as a unit of meaning), was coded exclusively to one code. Two researchers coded this content, a Kappa calculation performed to test reliability (κ) was .839 (*p* < .0005), representing a strong agreement [33]. The frequencies of these codes were then compared to the reported timing of offering first support to explore if there was a relationship.

### Participant characteristics

Of the 352 participants, 288 (81.8%) were women, mean age was 51.5 years (SD 9.9). Mean years in practice was 13.1 (SD 9.5) and mean treatments given per/week were 17.9 (SD 10.3). Further data in Table 1.

**Table 1:** Participant characteristics

**Continuous Variables**	**N**	**Min**	**Max**	**Mean**	**SD**
Age	352	26	74	51.46	9.86
Treatments p/week	352	2	80	17.93	10.24
Years in Practice	352	0	42	13.08	9.46
**Categorical Variables**			**Frequency (%)**		
Gender/Sex					
Female			288 (81.8%)		
Male			57 (16.2%)		
Other/Prefer not to say			7 (2%)		
Clinical Settings (not mutually exclusive)			352		
Have premises at home			142 (40.3%)		
Multidisciplinary clinic			135 (38.4%)		
Rent a treatment room			120 (34.1%)		
Provide home visits			73 (20.7%)		
Acupuncture clinic			66 (18.8%)		
Multi-bed clinic			32 (9.1%)		
Volunteer			17 (4.8%)		
NHS			7 (2%)		
Other			23 (6.5%)		
Main style of practice			352		
TCM			164 (46.6%)		
5 Element			79 (22.4%)		
Mix of Styles			59 (16.8%)		
Stems and Branches			7 (2%)		
Classical			7 (2%)		
Japanese			6 (1.7%)		
8 Principles			5 (1.4%)		
Tan			3 (0.9%)		
Six Channels			3 (0.9%)		
Other			6 (1.7%)		

## Results

### Prevalence and patterns of support for LHBC

#### Prevalence of support for LHBC

57.7% (*n* = 203/352) of participants reported offering LHBC support during their most recent consultation. Additionally 85.5% (*n* = 301/352) reported they had previously offered LHBC support to the last patient they saw*.* Participants reported that at the last appointment the following issues were supported: diet 32.4% (*n* = 114), physical activity 30.4% (*n* = 107), sleep hygiene 17.0% (*n* = 60), alcohol 5.7% (*n* = 20), smoking 3.7% (*n* = 13) and other lifestyle issue 25.3% (*n* = 89).

#### Patterns of support for LHBC in typical practice

**Fig. 1:**
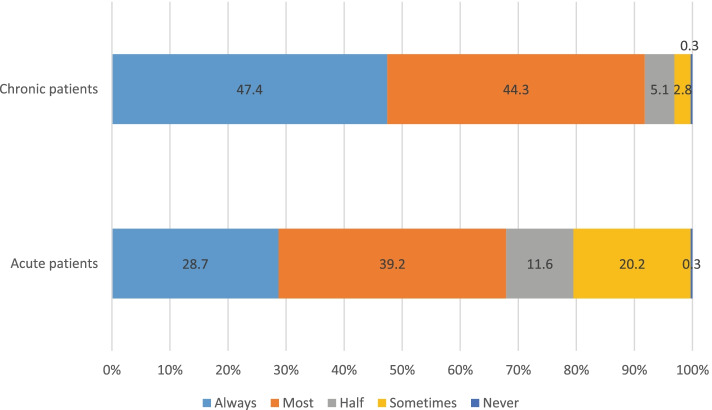
Pattern of Typical Support for Patients with Chronic and Acute Conditions. Legend: Self-reported frequency of typical support for lifestyle and health behaviour changes for patients presenting with chronic or acute conditions

Participants reported typical support for LHBC, i.e., supporting LHBC ‘most/all of the time’, was higher for patients with chronic conditions (91.7%, *n* = 323) than those with acute conditions (67.9%, *n* = 239) and this association was statistically significant *X*^*2*^ (4, *n* = 352) = 72.2601, *p* = < 0.001 (see Fig. 1).

Participants reported supporting change in diet, physical activity and sleep hygiene more than alcohol and smoking in typical practice for both chronic/acute conditions. For chronic conditions practitioners reported: diet 95.2% (*n* = 335), physical activity 93.2% (*n* = 328), sleep hygiene 87.5% (*n* = 308), alcohol 76.1% (*n* = 268) and smoking 69.3% (*n* = 244). For acute this was: physical activity 85.8% (*n* = 302), diet 83.5% (*n* = 294), sleep hygiene 71.3% (*n* = 251), alcohol 55.7% (*n* = 196) and smoking 44.6% (*n* = 157).

Content qualitative analysis of open text answers regarding ‘other lifestyle issues supported’ identified key additional areas of LHBC as: *Emotional/Cognitive regulation* (these were often specific practices such as relaxation techniques and meditation); *Stress management through daily life* (these included making changes to work, rest and social life*)*; and *Specific self-care treatments* (such as rehabilitation exercise) however this last area was usually a more explicit aspect of treatment rather than a behaviour. See Table 2.

**Table 2:** Additional areas of lifestyle/health behaviour change supported by acupuncturists (content analysis of open text responses)

Main Areas	Sub-categories	Quote
Emotional/Cognitive Regulation. Many of the additional areas of lifestyle change were related to methods of emotional/cognitive regulation. Some of these can clearly be considered as lifestyle behaviours (e.g. adopting a regular meditation practice) but some were specific cognitive strategies (e.g. changing mind-set)	Relaxation/Stress Reduction techniques. Specific practices which aim to reduce the individuals’ perception or experience of stress (distinct from avoiding stress or generally relaxing).	*“Relaxation techniques as he suffers with stress related migraine”*
	Meditation/Mindfulness	*“I sometimes suggest they look at using apps such as Insight Timer for guided meditations or other tools that may help them to manage their symptoms”*
	Qigong, breathing or yoga practices	*“… eventually settling on qigong as a calming practice he can do on his own”*
	Mind-set/Cognitive strategies	*“And changing negative thought patterns - encouraging people to be kinder to themselves and see the good in what they do rather than focusing on what they do wrong”* *“… constructive re-framing of thoughts and ideas”*
Stress Management through Daily Life Patterns Another set of lifestyle changes related to making adjustments to everyday life, such as work, relationships and rest, in order to reduce psychosocial stress.	Work (or work/life balance)	*“Work issues, maybe needing time off or cutting down.”* *“Reducing their workload and subsequent stress levels”*
	Relationships Change to social life/difficult personal relationships	*“Patient cares for a very disabled daughter which is very stressful for her. Talked with her about having some respite care for the daughter.”* *“To include fun and social contact in their life.”*
	Rest General time for self, for rest and relaxation	*“A lot of patients leading very busy lifestyles and do not rest properly. Help try and emphasise the importance of rest”*
	Hobbies	*“She was very aware of diet and exercise. I recommended art classes to help with relaxation and mood”* *“Find something that make them relaxed and happy and do it regularly”*
	Time outdoors/in nature	*“getting out at lunch time to get some daylight and fresh air as the evenings draw in.”*
	Reduce use of tech/screens	*“Increasingly, reduction in time online and general screen use.”*
Self-Care Treatments This set of lifestyle changes were very specific self-care practices which were often adopted as methods for treating a condition (likely temporarily).	Postural exercise, rehabilitation exercises and repetitive strain corrections	*“posture and positioning when using computer”* *“I often recommend pelvic floor muscle training to help with movement and bladder problem”*
	Products and other treatments (e.g. herbal teas, supplements, self-massage)	*“Abdominal massage, warming foods and teas”*
	Temperature regulation (protection from cold/wind/heat/damp)	*“Moving from a very damp house.”*

### Barriers and facilitators to engaging in support for lifestyle and health behaviour change (in acute presentations)

Scale scores of participants’ attitudes, PBC, norms and other characteristics (use of behaviour change techniques, personal lifestyle habits and demographic data) were compared with reported frequency of supporting LHBC in acute patients (Table 3).

**Table 3:** Participants Attitudes, PBC, Norms, Use of BCTs and Personal Lifestyle Habit Scores (Descriptive Statistics)

Predictor Variables	N	Min	Max (Max Possible)	Mean	SD
Attitudes to clinical role	352	1.0	4.4 (7.0)	1.8	0.62
Attitudes to behaviours	352	1.0	5.0 (5.0)	1.68	0.56
Perceived Behavioural Control	352	1.0	7.0 (7.0)	2.60	1.01
Norms	352	1.0	5.3 (7.0)	2.35	0.88
BCT usage (total)	352	0.0	20.0 (20.0)	12.09	4.58
Personal lifestyle habits (optional section original data only)	236	2.0	8.0 (8.0)	6.47	1.18

#### Regression analysis

Simple univariate regression analysis found higher scores in PBC, Attitude to Behaviours, Attitude to Clinical Role and Norms were positively associated with providing LHBC support to patients with acute conditions. Higher scores in Total BCTs reported, Treatments p/week, Volunteer Setting, and Five-element Style were slightly negatively associated with LHBC support (acute conditions) (*p* < 0.05). See Table 4.

**Table 4:** Predictors of Support for Lifestyle/Health Behaviour Change Support (acute presentations): Univariate Linear Regression

Predictor Variables	B	SE	β	t	Sig
Attitudes to clinical role	0.592	0.062	0.339	9.523	0.000
Attitudes to behaviours	0.643	0.070	0.331	9.225	0.000
Perceived Behavioural Control	0.405	0.038	0.377	10.786	0.000
Social Norms	0.271	0.045	0.221	5.972	0.000
BCT usage (total)	−0.069	0.009	−0.290	−8.038	0.000
Treatments p/week	− 0.013	0.004	− 0.125	−3.293	0.001
Age	−0.006	0.004	− 0.051	−1.333	0.183
Years in practice	−0.003	0.004	−0.030	−0.791	0.429
Setting:					
Multidisciplinary clinic	−0.067	0.084	−0.030	−0.799	0.424
Have own premises at home	−0.039	0.083	−0.018	−0.465	0.642
Provide home visits	0.093	0.101	0.035	0.920	0.358
Rent a room	−0.164	0.086	−0.072	−1.904	0.057
Acupuncture clinic	0.113	0.105	0.041	1.078	0.281
Multibed clinic	−0.059	0.142	−0.016	− 0.417	0.677
NHS	0.337	0.293	0.043	1.149	0.251
Volunteer	−0.377	0.190	−0.075	−1.981	0.048
Other	0.160	0.166	0.037	0.968	0.333
Main style of practice:					
TCM	0.084	0.082	0.039	1.029	0.304
Eight Principles	1.175	0.343	0.128	3.424	0.001
Five Element	−0.230	0.098	−0.088	−2.350	0.019
Japanese	−0.415	0.316	−0.050	−1.314	0.189
Other	0.093	0.316	0.011	0.295	0.768
Mix	−0.025	0.110	−0.009	− 0.232	0.817
Tan	0.093	0.446	0.008	0.208	0.835
Stems & Branches	0.337	0.293	0.043	1.149	0.251
Classical	−0.101	0.293	−0.013	−0.343	0.732
Six Channel	0.429	0.445	0.036	0.963	0.336
Personal lifestyle score	−0.034	0.042	−0.037	− 0.797	0.426

Table 4 Legend: Results of univariate linear regressions assessing relationships between each predictor alone and the outcome variable (providing support for LHBC to patient presenting with acute conditions). Scores with a negative figure indicate a reverse correlation, i.e. higher score predicted less frequent support

A hierarchical multiple linear regression analysis was carried out using the 8 significant single predictor variables from the univariate analysis. The multiple regression revealed that at Stage one, Attitude to Behaviours contributed significantly to the regression model, F (1,347) = 38.22 *p* < .01) and accounted for 9.7% of the variation in Acute Support. Introducing the Attitude to Clinical Role variable explained an additional 5.5% of variation and this change in R^2^ was significant, F (1,346) = 22.70 *p* < .001. Adding PBC to the regression model explained an additional 6.6% of the variation in Acute Support and this change in R^2^ was significant, F (1,345) = 29.701 *p* < .001. The addition of Norms to the regression model was not significant, F (1,344) = 3.09, *p* = .080. The addition of Setting Volunteer was not significant F (1, 343) = 1.25 *p* = .263; the addition of Five Element Style was not significant F (1, 342) = 1.29 *p* = .257. The addition of BCT Usage (Total) explained an additional 1.3% of the variance this change in R^2^ was significant, F (1,341) = 5.84 *p* < .005. The addition of Treatments p/week was not significant F (1,340) = 1.05 *p* = .306). A significant regression equation was found for the overall Model F (8,340) = 14.048, *p* < 0.001), which explained 24.8% of the variance (adjusted *R*^*2*^ = 0.248) in LHBC support scores (acute conditions). See Table 5.

**Table 5:** Predictors of lifestyle/health behaviour change support in acute presentations - multivariate regression analysis

Predictor Variables	Co-efficient (standardised)	95% CI	*p*
Attitudes to health behaviours	0.197	0.193;0.605	0.000
Attitudes to clinical role	0.095	−0.032;0.366	0.100
Perceived Behavioural Control (PBC)	0.229	0.132;0.356	0.000
Norms	0.099	−0.004;0.249	0.057
Volunteer setting	−0.043	−0.690;0.261	0.375
Five Element style acupuncture	−0.052	−0.375;0.108	0.276
BCT usage total	−0.117	−0.052;-0.004	0.021
Treatments p/week	−0.050	−0.017;0.005	0.306

Table 5 Legend: Results of multivariate linear regression assessing relationships between eight predictors and the outcome variable (providing support for LHBC to patients presenting with acute conditions). Scores with a negative figure indicate a reverse correlation, i.e. higher score predicted less frequent support.

This analysis shows that traditional acupuncturists were more likely to report offering LHBC support for acute conditions when they believed health behaviours were important for health, when they felt capable of offering support, and when they believed that promoting health behaviour was part of their clinical work. They were slightly less likely to support LHBC if they reported using a greater number of different BCTs.

### What guides acupuncturists’ decisions about when to first offer lifestyle change support?

The most frequent reasons given for deciding when to first offer support for LHBC were: perceived patient receptiveness; whether the presenting condition/diagnosis would likely respond to LHBC; and whether a good relationship was established with the patient (See Table 6).

**Table 6:** Acupuncturist’s reasons for deciding when to first offer support for lifestyle/health behaviour changes

Reason	Definition	Frequencies	%
Patient Receptiveness	Responds to the patient’s readiness for change (openness/commitment/request)	129	36.6
Depends on Diagnosis	Depends on the diagnosis/condition including the cause of the condition (aetiology) or likely impact of behaviour change on the condition	77	21.9
After Relationship Established	Forming a good relationship and rapport with the patient needs to be established first.	76	21.6
Standard Practice	Always gives support for lifestyle change as standard part of practice (usually comes up in initial case history or traditional diagnosis)	60	17
Gentle Approach	Introduces support for lifestyle change in a step by step, gentle way starting with small changes	34	9.7
Patient Capability	Patient’s capability to make appropriate lifestyle changes/how difficult the changes will be to make (more than just readiness or openness – indicates something about whether the patient actually can make changes)	23	6.5
Guided by Conversation	Guided by a two-way discussion and conversation with patient	20	5.7
Wait Treatment Response (sub-categories a, b, c below):	Waits to see how well acupuncture treatment is working	16	4.5
a) not working	if condition not improving with acupuncture	4	1.1
b) after working	after condition has improved with acupuncture	10	2.8
c) general	unspecified wait for treatment response	2	0.6
Receptive at First	Believes patients are generally looking for change when they come or believes patients are usually most receptive to making changes on their first visit.	13	3.7
First Treatment Full	Feels the first appointment is too soon to offer lifestyle change support (already full of information, too overwhelming at first).	11	3.1
Intuition	Spontaneous gut feeling or whenever the idea comes to them (Intuition/Experience/Right moment /When I think)	9	2.6
General Health Behaviours	Whether health behaviour needs general improvement (not for specific condition)	8	2.3
If Patient Long-term	Offers support if patient is long term	4	1.1
After Time to Consider	Needs enough time to gather information and consider diagnosis/appropriate recommendations before offering any lifestyle change support	3	9
Time Available	Offers support if there is time available	2	0.6

A chi-square test of independence was performed to examine the relation between these reasons and the reported timing of first offering support (at or after first visit). Those being guided by the establishment of a good relationship were less likely to offer support at first visit, *X*2 (1,*N* = 261) = 4.7, *p* = .030. Those being guided by standard practice were more likely to offer support at first visit, *X*2 (1,*N* = 261) = 4.4,*p* = .035.

## Discussion

This was the first nationwide survey of UK acupuncturists on lifestyle/health behaviour support and has found robust evidence that acupuncturists in the UK are already participating in a high level of support for promoting healthy lifestyles. 57.7% of most recent patient visits included some LHBC support. Reported typical support for LHBC was very high for chronic presentations (91.7% most/all of the time) and lower (though still fairly high) with acute presentations (67.9% most/all of the time). Practitioners were more likely to report supporting LHBC in acute presentations when they had more positive attitudes towards and felt better able to offer support for behaviour change. These findings fit with our previous work suggesting traditional acupuncturists support LHBC more frequently for chronic conditions [15]. This could be due to practitioners’ perception of the greater impact of health behaviours on chronic conditions or because potentially longer treatment programmes with long-term, chronic patients facilitate a strong therapeutic relationship (which many practitioners prefer to establish before raising LHBC).

We have found that physical activity and diet changes are most widely supported by UK acupuncturists, followed by sleep hygiene, alcohol and smoking. The relatively lower level of support for smoking and alcohol suggests there may be a training need for acupuncturists in this area. It is possible that current public health goals (e.g. in relation to smoking and alcohol consumption) are not widely taught in acupuncture training programmes which may rely on more traditional lifestyle advice. There is scope for updating some traditional lifestyle recommendations in line with changes in population characteristics over time. However this finding is similar to findings in other CM therapies, e.g. the level of chiropractors’ support for smoking and alcohol reduction was similar to medical health professionals (but the support for other behaviours, such as physical activity was higher) [34, 35].

We identified wide reporting of additional aspects of lifestyle change, including emotional/cognitive regulation; stress reduction in daily life and specific self-care treatments. The support for behaviours which address psychosocial stress is perhaps partly due to the holistic/biopsychosocial model adopted by traditional acupuncturists and has been found in other CM therapies [35]. Addressing psychosocial stress through behaviour change may be an important area of future research, particularly in light of evidence for the social determinants of health, some of which (though not all) are mediated through psychosocial stress [36].

Our finding that acupuncturists’ decisions regarding when to first offer lifestyle change support were guided by; perceived patient receptiveness, whether presenting diagnosis/condition will improve with LHBC and whether a good relationship is established with the patient, provides insight into acupuncturists judgements concerning the circumstances which may facilitate successful behaviour change outcomes. Across other health professions some studies have found that strong therapeutic alliance is associated with engagement in weight management [37] and adherence to physiotherapy exercise programmes and physical activity [38]. The place of therapeutic relationships in behaviour change models has not yet been fully developed and the role of therapeutic alliance in health behaviour change needs further research. Our findings suggest a tension between the practice of traditional acupuncturists and the Make Every Contact Count strategy widely adopted in the UK’s NHS. This strategy encourages frequent, repeated reminders about health behaviours at each contact with health professionals. Our evidence in the context of traditional acupuncture practice however suggests it may be important to establish a good relationship with patients before raising LHBC, taking an approach which is guided more by cues from patients and linking advice to patients’ concerns. More evidence is needed to understand which approaches are most effective in different contexts and different patient groups. This work would also need to include the development of suitable measures of LHBC patient outcomes in acupuncture.

Overall these findings suggest that traditional acupuncturists are already working in line with key public health aims for improving health behaviours. While there may be scope for increasing support within acute presentations our findings suggest that many acupuncturists believe more effective lifestyle change support occurs when patients are receptive, advice is linked to presenting condition and a good relationship is established.

### Strengths and limitations

Our findings for prevalence of LHBC support is highly reliable, using a snapshot measure to reduce bias in recall or misreporting in case notes. Our sample size was statistically powerful and representative of the major association of traditional acupuncturists in the UK, and participant characteristics reflected characteristics of the overall population (with 8% more female respondents in sample). However, the findings may be culturally specific since traditional acupuncturists in the UK may have developed specific values and attitudes towards LHBC. Though rooted in traditional Chinese/East Asian medicine, the UK acupuncturist population’s norms and styles of practice may vary significantly from those in China and East Asia (and other regions). Also traditional acupuncture practised as a complex intervention differs from more narrowly defined styles of acupuncture (i.e. medical acupuncture or ‘dry’ needling) and it is likely that these findings will not apply to those providers. Our measures of typical support (for acute and chronic conditions) rely on acupuncturist self-reports and may suffer from recall bias. However alternative methods for assessing typical support (e.g. using case notes) may also result in bias (under/misreporting of discussions in written notes). We have not explored in detail the utilisation of different BCTs by acupuncturists, there may be scope for understanding and improving use of techniques such as goal setting, monitoring and action-planning.

## Conclusions

This study shows that acupuncturists’ support for LHBC for patients presenting with chronic conditions is high. There is some scope for increasing support for LHBC for patients presenting with acute conditions, (i.e. through enhancing attitudes towards the importance of health behaviours on health outcomes and increasing acupuncturists’ confidence in their ability to support LHBC). However the emphasis acupuncturists place on the importance of the therapeutic relationship, patient receptiveness and linking advice to symptoms suggests there may be important differences between acupuncture practice and mainstream healthcare (e.g. high level of contact, visits of long duration, holistic/biopsychosocial approach) which impact the mechanisms of action of behaviour change. Lifestyle change support may be most effective in this context when there is a strong relationship or a clear purpose (i.e. improvement of current symptoms) for working together on lifestyle change. More evidence is needed to understand patient perspectives regarding this approach and to integrate these findings with behaviour change models.

## Supplementary Information

**Additional file 1: Appendix 1.** Downloaded Version of Questionnaire.

**Additional file 2: Appendix 2.** Development of TPB Scales [39, 40].

## Data Availability

The datasets generated and/or analysed during the current study are not publicly available as we do not have consent to share the data.

## Abbreviations

BAcC: British Acupuncture Council
BCT: Behaviour Change Techniques
CIS: Critical Interpretive Synthesis
CM: Complementary Medicine
LHBC: Lifestyle and Health Behaviour Change
NHS: National Health Service
PBC: Perceived Behavioural Control
PSA: Professional Standards Authority
TPB: Theory of Planned Behaviour
UK: United Kingdom
VIF: Variance Inflation Factor

## References

[1] Steel N, Ford JA, Newton JN, Davis ACJ, Vos T, Naghavi M, et al. Changes in health in the countries of the UK and 150 English local authority areas 1990–2016: a systematic analysis for the global burden of disease study. Lancet. 2018. 10.1016/S0140-6736(18)32207-4. 10.1016/S0140-6736(18)32207-4 PMC6215773 30497795

[2] Garcia-Perdomo HA, Zapata-Copete J, Rojas-Ceron CA. Sleep duration and risk of all cause mortality: a systematic review and meta-analysis. Epidemiol Psychiatr Sci. 2018. 10.1017/S2045796018000379. 10.1017/S2045796018000379 PMC6998920 30058510

[3] Royal Society for Public Health (2015). Rethinking the wider public health workforce.

[4] Hawk C, Ndetan H, Evans MW Jr. Potential role of complementary and alternative health care providers in chronic disease prevention and health promotion: an analysis of National Health Interview Survey data. Prev Med. 2012. 10.1016/j.ypmed.2011.07.002. 10.1016/j.ypmed.2011.07.002 21777609

[5] The Professional Standards Authority and the Royal Society for Public Health (2017). Untapped Resources: Accredited Registers in the Wider Workforce.

[6] Davis MA, West AN, Weeks WB, Sirovich BE. Health behaviors and utilization among users of complementary and alternative medicine for treatment versus health promotion. Health Serv Res. 2011. 10.1111/j.1475-6773.2011.01270.x. 10.1111/j.1475-6773.2011.01270.x PMC3207184 21554272

[7] Williams-Piehota PA, Sirois FM, Bann CM, Isenberg KB, Walsh EG (2011). Agents of change: how do complementary and alternative medicine providers play a role in health behavior change?. Altern Ther Health Med.

[8] Bishop FL, Lauche R, Cramer H, Pinto JW, Leung B, Hall H (2019). Health behavior change and complementary medicine use: National Health Interview Survey 2012. Medicina..

[9] McFadden KL, Hernández TD, Ito TA (2010). Attitudes toward complementary and alternative medicine influence its use. Explore..

[10] MacPherson H, Thorpe L, Thomas K (2006). Beyond needling - therapeutic processes in acupuncture care: a qualitative study nested within a low-back pain trial. J Altern Complement Med.

[11] Hopton AK, Curnoe S, Kanaan M, MacPherson H (2012). Acupuncture in practice: mapping the providers, the patients and the settings in a national cross-sectional survey. BMJ Open.

[12] Austin S, Ramamonjiarivelo Z, Qu H, Ellis-Griffith G (2015). Acupuncture use in the United States: who, where, why, and at what Price?. Health Mark Q.

[13] Upchurch DM, Rainisch BW (2014). A sociobehavioral wellness model of acupuncture use in the United States, 2007. J Altern Complement Med.

[14] Gressel Raz O, Samuels N, Levy M, Leviov M, Lavie O, Ben-Arye E (2020). Association between physical activity and use of complementary medicine by female oncology patients in an integrative palliative care setting. J Altern Complement Med.

[15] Pinto JW, Bradbury K, Newell D, Bishop FL. Lifestyle and health behavior change in traditional acupuncture practice: a systematic critical interpretive synthesis. J Altern Complement Med. 2021. 10.1089/acm.2020.0365. 10.1089/acm.2020.0365 33332183

[16] Nik Nabil WN, Zhou W, Shergis JL, Mansu S, Xue CC, Lin ZA (2015). Management of respiratory disorders in a Chinese medicine teaching clinic in Australia: review of clinical records. Chin Med.

[17] Cheshire A, Polley M, Peters D, Ridge D (2013). Patient outcomes and experiences of an acupuncture and self-care service for persistent low back pain in the NHS: a mixed methods approach. BMC Complement Altern Med.

[18] Cochrane S, Smith CA, Possamai-Inesedy A, Bensoussan A. Prior to Conception: The role of an acupuncture protocol in improving Women’s reproductive functioning assessed by a pilot pragmatic randomised controlled trial. Evid Based Complement Alternat Med. 2016;2016:3587569. 10.1155/2016/3587569 PMC4868913 27242910

[19] MacPherson H, Elliot B, Hopton A, Lansdown H, Birch S, Hewitt C (2017). Lifestyle advice and self-care integral to acupuncture treatment for patients with chronic neck pain: secondary analysis of outcomes within a randomized controlled trial. J Altern Complement Med.

[20] Borud EK, Alraek T, White A, Grimsgaard S (2010). The acupuncture on hot flashes among menopausal women study: observational follow-up results at 6 and 12 months. Menopause.

[21] Evans M, Paterson C, Wye L, Chapman R, Robinson J, Norton R (2011). Lifestyle and self-care advice within traditional acupuncture consultations: a qualitative observational study nested in a co-operative inquiry. J Altern Complement Med.

[22] Rugg S, Paterson C, Britten N, Bridges J, Griffiths P (2011). Traditional acupuncture for people with medically unexplained symptoms: a longitudinal qualitative study of patients’ experiences. Br J Gen Pract.

[23] Bandura A (1986). Social foundations of thought and action.

[24] Armitage CJ, Conner M (2001). Efficacy of the theory of planned behaviour: a meta-analytic review. Br J Soc Psychol.

[25] Steptoe A, Doherty S, Kendrick T, Rink E, Hilton S (1999). Attitudes to cardiovascular health promotion among GPs and practice nurses. Fam Pract.

[26] McAvoy BR, Kaner EF, Lock CA, Heather N, Gilvarry E (1999). Our healthier nation: are general practitioners willing and able to deliver? A survey of attitudes to and involvement in health promotion and lifestyle counselling. Br J Gen Pract.

[27] Litaker D, Flocke SA, Frolkis JP, Stange KC. Physicians’ attitudes and preventive care delivery: insights from the DOPC study. Prev Med. 2005. 10.1016/j.ypmed.2004.07.015. 10.1016/j.ypmed.2004.07.015 15749138

[28] Maujean A, Kendall E, Ehrlich C, Kisely S. The capacity for health promotion survey. Gen Hosp Psychiatry. 2014. 10.1016/j.genhosppsych.2014.08.007. 10.1016/j.genhosppsych.2014.08.007 25204778

[29] Michie S, Richardson M, Johnston M, Abraham C, Francis J, Hardeman W (2013). The behavior change technique taxonomy (v1) of 93 hierarchically clustered techniques: building an international consensus for the reporting of behavior change interventions. Ann Behav Med.

[30] Godwin M, Streight S, Dyachuk E, van den Hooven C, Ploemacher J, Seguin R (2008). Testing the simple lifestyle Indicator questionnaire: initial psychometric study. Can Fam Physician.

[31] Willis GB, Artino AR Jr. What do our respondents think we’re asking? Using cognitive interviewing to improve medical education surveys. J Grad Med Educ. 2013. 10.4300/JGME-D-13-00154.1. 10.4300/JGME-D-13-00154.1 PMC3771159 24404294

[32] Sainani KL (2015). Dealing With Missing Data.PM & R.

[33] McHugh ML (2012). Interrater reliability: the kappa statistic. Biochem Med.

[34] Ndetan HT, Bae S, Evans MW, Rupert RL, Singh KP. Characterization of health status and modifiable risk behavior among United States adults using chiropractic care as compared with general medical care. J Manipulative Physiol Ther. 2009. 10.1016/j.jmpt.2009.06.012. 10.1016/j.jmpt.2009.06.012 19712783

[35] Fikar PE, Edlund KA, Newell D. Current preventative and health promotional care offered to patients by chiropractors in the United Kingdom: a survey. Chiropr Man Therap. 2015. 10.1186/s12998-015-0053-z. 10.1186/s12998-015-0053-z PMC4353665 25755875

[36] Braveman P, Gottlieb L. The social determinants of health: It’s time to consider the causes of the causes. Public Health Rep. 2014. 10.1177/00333549141291S206. 10.1177/00333549141291S206 PMC3863696 24385661

[37] Sturgiss EA, Sargent GM, Haesler E, Rieger E, Douglas K. Therapeutic Alliance and obesity management in primary care - a cross-sectional pilot using the working Alliance inventory. Clin Obes. 2016. 10.1111/cob.12167. 10.1111/cob.12167 27863074

[38] Moore AJ, Holden MA, Foster NE, Jinks C. Therapeutic alliance facilitates adherence to physiotherapy-led exercise and physical activity for older adults with knee pain: a longitudinal qualitative study. J Physiother. 2020. 10.1016/j.jphys.2019.11.004. 10.1016/j.jphys.2019.11.004 31843425

[39] Kaiser HF, Rice J. Little Jiffy, Mark IV. Educ Psychol Meas. 1974. 10.1177/001316447403400115.

[40] Field A (2013). Discovering statistics using IBM SPSS statistics.

