# Supplementary material for: Lifestyle and health behaviour change support in traditional acupuncture: a mixed method survey study of reported practice (UK)
Source: BMC Complement Med Ther. 2022 Sep 21;22:248. doi: 10.1186/s12906-022-03719-6 (PMC9490899; doi:10.1186/s12906-022-03719-6)
Supplement: Supplementary file 1 — Additional file 1: Appendix 1. Downloaded Version of Questionnaire. [file 12906_2022_3719_MOESM1_ESM.docx]

Appendix 1: Downloaded Version of Questionnaire

Traditional acupuncture and lifestyle change. What do you do in your clinic?

- I agree (1)
- No thanks (2)

End of Block: Landing page

Start of Block: Eligibility check

Q2 Are you currently a registered practicing member of the British Acupuncture Council?

- Yes (1)
- Don't know (2)
- No (3)

End of Block: Eligibility check

Start of Block: Prevalence of lifestyle work

Q3 Do you typically try to help your patients change their lifestyle - including smoking, diet/eating habits, physical activity, sleep hygiene* and alcohol consumption?   

 *Sleep hygiene refers to the habits and practices that are conducive to sleeping well on a regular basis

- Yes (1)
- No (3)
- Any comments (4) ________________________________________________

| Page Break |  |
| --- | --- |

Q4 Thinking now about patients who are seeking help for acute* problems. How frequently do you try to help these patients change their lifestyle (including smoking, diet/eating habits, physical activity, sleep hygiene and alcohol consumption)?
*Acute problems are any type of condition which has a rapid onset and short duration i.e. less than 3 months

- Always (1)
- Most of the time (2)
- About half the time (3)
- Sometimes (4)
- Never (5)

Q5 In treatments for acute problems which of the issues below would you typically try to help your patients change ? Tick as many as apply.

- Alcohol consumption (1)
- Diet / eating habits (2)
- Sleep hygiene (3)
- Smoking (if patient smokes) (4)
- Physical activity (5)
- Other, please provide details (6) ________________________________________________

| Page Break |  |
| --- | --- |

Q6
Thinking now about patients who are seeking help for chronic* problems. How frequently do you try to help these patients change their lifestyle (including smoking, diet/eating habits, physical activity, sleep hygiene and alcohol consumption)? 

*Chronic problems are any type of condition where symptoms are long-lasting i.e. more than 3 months

- Always (1)
- Most of the time (2)
- About half the time (3)
- Sometimes (4)
- Never (5)

Q7 In treatments for chronic problems which of the issues below would you typically try to help your patients change? Tick as many as apply.

- Alcohol consumption (1)
- Sleep hygiene (2)
- Smoking (if patient smokes) (3)
- Diet and eating habits (4)
- Physical activity (5)
- Other, please provide details (6) ________________________________________________

Q8 Typically when do you FIRST try to help your patients change their lifestyle? Tick one answer.

- At first visit (1)
- After first visit but within 2 weeks of first visit (2)
- Within 2-4 weeks of first visit (3)
- Within 4-6 weeks of first visit (4)
- Within 6 -8 weeks of first visit (5)
- More than 8 weeks after first visit (6)
- Don't know / No typical pattern (7)
- Never (8)

Q9 What usually guides your decision about when to first try to help your patients change their lifestyle? Please use your own words below (optional):

________________________________________________________________

________________________________________________________________

________________________________________________________________

________________________________________________________________

________________________________________________________________

| Page Break |  |
| --- | --- |

Q10 Thinking about the last patient you treated. Have you EVER tried to help this patient change their lifestyle?

- Yes (1)
- Maybe (2)
- No (3)

Q11 Which areas of lifestyle have you EVER tried to help this patient change? Tick as many as apply.

- Diet / eating habits (1)
- Physical activity (2)
- Sleep hygiene (3)
- Alcohol consumption (4)
- Smoking (if patient smokes) (5)
- Other, please provide details (6)
- Don't know / Can't remember (7)

Q12 Again thinking about the last patient you treated. Did you try to help this patient change their lifestyle at that LAST visit?

- Yes (1)
- Maybe (2)
- No (3)

Q13 Which areas of lifestyle did you try to help this patient change at the LAST visit? Tick as many as apply.

- Diet / Eating habits (1)
- Physical activity (2)
- Sleep hygiene (3)
- Alcohol consumption (4)
- Smoking (if patient smokes) (5)
- Other, please provide details (6) ________________________________________________
- Don't know / Can't remember (8)

End of Block: Prevalence of lifestyle work

Start of Block: Attitudes: Importance of lifestyle issues for health

Q14 How important do you believe each of the following 5 lifestyle issues are for health? Please tick boxes that apply.

|  | Extremely important (1) | Very important (2) | Moderately important (3) | Slightly important (4) | Not at all important (5) | Don't Know (6) |
| --- | --- | --- | --- | --- | --- | --- |
| Smoking / Not smoking (1) |  |  |  |  |  |  |
| Physical activity (2) |  |  |  |  |  |  |
| Diet and eating habits (3) |  |  |  |  |  |  |
| Alcohol consumption (4) |  |  |  |  |  |  |
| Sleep hygiene (5) |  |  |  |  |  |  |

End of Block: Attitudes: Importance of lifestyle issues for health

Start of Block: Perceived behavioural control for promoting lifestyle change

Q15 Please indicate how much you agree or disagree with the following statements:


I have no time to spend on lifestyle change with patients

- Strongly agree (1)
- Agree (2)
- Somewhat agree (3)
- Neither agree nor disagree (4)
- Somewhat disagree (5)
- Disagree (6)
- Strongly disagree (7)

Q16
 I am confident in my ability to help patients change their lifestyle habits.

- Strongly agree (1)
- Agree (2)
- Somewhat agree (3)
- Neither agree nor disagree (4)
- Somewhat disagree (5)
- Disagree (6)
- Strongly disagree (7)

Q17 I feel properly trained to help patients make lifestyle changes

- Strongly agree (1)
- Agree (2)
- Somewhat agree (3)
- Neither agree nor disagree (4)
- Somewhat disagree (5)
- Disagree (6)
- Strongly disagree (7)

Q18
I find it difficult to help patients make lifestyle changes

- Strongly agree (1)
- Agree (2)
- Somewhat agree (3)
- Neither agree nor disagree (4)
- Somewhat disagree (5)
- Disagree (6)
- Strongly disagree (7)

| Page Break |  |
| --- | --- |

End of Block: Perceived behavioural control for promoting lifestyle change

Start of Block: Attitudes: to lifestyle change work

Q19
Please indicate how much you agree or disagree with the following statements:


Finding out about patients' physical activity is a very important part of my work.

- Strongly agree (1)
- Agree (2)
- Somewhat agree (3)
- Neither agree nor disagree (4)
- Somewhat disagree (5)
- Disagree (6)
- Strongly disagree (7)

Q20
Finding out about patients' smoking habits is a very important part of my work.

- Strongly agree (1)
- Agree (2)
- Somewhat agree (3)
- Neither agree nor disagree (4)
- Somewhat disagree (5)
- Disagree (6)
- Strongly disagree (7)

Q21
Finding out about patients' alcohol consumption is a very important part of my work.

- Strongly agree (1)
- Agree (2)
- Somewhat agree (3)
- Neither agree nor disagree (4)
- Somewhat disagree (5)
- Disagree (6)
- Strongly disagree (7)

Q22
Finding out about patients' sleep hygiene is a very important part of my work.

- Strongly agree (1)
- Agree (2)
- Somewhat agree (3)
- Neither agree nor disagree (4)
- Somewhat disagree (5)
- Disagree (6)
- Strongly disagree (7)

Q23
Finding out about patients' diet/eating habits is a very important part of my work.

- Strongly agree (1)
- Agree (2)
- Somewhat agree (3)
- Neither agree nor disagree (4)
- Somewhat disagree (5)
- Disagree (6)
- Strongly disagree (7)

Q24
My work is to provide treatment, I leave lifestyle change to others.

- Strongly agree (1)
- Agree (2)
- Somewhat agree (3)
- Neither agree nor disagree (4)
- Somewhat disagree (5)
- Disagree (6)
- Strongly disagree (7)

| Page Break |  |
| --- | --- |

End of Block: Attitudes: to lifestyle change work

Start of Block: Norms re lifestyle change work

Q25 Please indicate how much you agree or disagree with the following statements:


My fellow acupuncturists would expect me to include lifestyle change in my treatments.

- Strongly agree (1)
- Agree (2)
- Somewhat agree (3)
- Neither agree nor disagree (4)
- Somewhat disagree (5)
- Disagree (6)
- Strongly disagree (7)

Q26 The acupuncture college I attended values working on lifestyle change within consultations.

- Strongly agree (1)
- Agree (2)
- Somewhat agree (3)
- Neither agree nor disagree (4)
- Somewhat disagree (5)
- Disagree (6)
- Strongly disagree (7)

Q27 Most traditional acupuncturists include lifestyle change alongside their treatments

- Strongly agree (1)
- Agree (2)
- Somewhat agree (3)
- Neither agree nor disagree (4)
- Somewhat disagree (5)
- Disagree (6)
- Strongly disagree (7)

End of Block: Norms re lifestyle change work

Start of Block: Techniques and Intervention Ingredients

Q28 Do you think that giving explanations for your lifestyle advice using traditional Chinese/East Asian medicine theory is important for helping patients to change lifestyle habits?

- Extremely important (1)
- Very important (2)
- Moderately important (3)
- Slightly important (4)
- Not at all important (5)

Q29 Do you think giving individualised lifestyle advice based on a specific diagnosis is important for helping patients to change lifestyle habits?

- Extremely important (35)
- Very important (36)
- Moderately important (37)
- Slightly important (38)
- Not at all important (39)

Q30 Do you think the effect of needling (or other acupoint stimulation) on symptoms such as pain, sleep, stress or nicotine withdrawal is important for helping patients to change lifestyle habits?

- Extremely important (1)
- Very important (2)
- Moderately important (3)
- Slightly important (4)
- Not at all important (5)

Q31 Do you think the effect of needling (or other acupoint stimulation) on patients' awareness of their own body sensations is important for helping patients to change lifestyle habits?

- Extremely important (1)
- Very important (2)
- Moderately important (3)
- Slightly important (4)
- Not at all important (5)

Q32 Do you think a strong therapeutic relationship between patient and practitioner is important for helping patients to change lifestyle habits?

- Extremely important (1)
- Very important (2)
- Moderately important (3)
- Slightly important (4)
- Not at all important (5)

Q33 Are there any other aspects of traditional acupuncture which you think are important for helping patients to change their lifestyle habits?

________________________________________________________________

________________________________________________________________

________________________________________________________________

________________________________________________________________

________________________________________________________________

|  |
| --- |

Q34 Do you typically use any of the following techniques to help patients make lifestyle changes?  Please tick all that apply.

- Provide instructions on a lifestyle issue (e.g. give written or verbal advice on diet) (3)
- Ask questions about patients' recent behaviour to remind them about lifestyle changes (23)
- Tell patient about health consequences of a positive lifestyle change (e.g. reducing sugar in diet will improve energy levels) (7)
- Ask patient to self-monitor lifestyle/behaviour (e.g. ask patient to keep a diary record of diet) (5)
- Suggest swapping an unwanted lifestyle behaviour with better one (e.g. suggest the patient walks instead of driving a car to increase physical activity) (9)
- Goal setting (help patient to set specific goal around a lifestyle change e.g. to walk a certain amount of steps each day or to eat 5 pieces of fruit/veg per day) (1)
- Reward positive lifestyle behaviour change (e.g. by congratulating or praising the patient) (11)
- Problem solving (help patient to look at the things that influence their lifestyle choices and choose ways to support change) (2)
- Help patient to change their perspective on a lifestyle behaviour (e.g. change their view of smoking as social to anti-social) (14)
- Advise pharmacological support (e.g. nicotine patches) (16)
- Action planning (help patient make a plan to do a specific lifestyle behaviour in a particular context e.g. to cycle to work on a Monday morning) (4)
- Advise the patient to seek social support in relation to a lifestyle change (e.g. doing physical activity with friends) (6)
- Use reminders to trigger patient to do a positive lifestyle behaviour (e.g. suggest post-it notes on the fridge to remind patient about healthy eating) (8)
- Habit formation (i.e. getting a patient to repeat a behaviour in the same context lots of times until it can be done automatically) (10)
- Self-incentives or rewards (e.g. patient gives themselves a reward if they stick to a lifestyle change) (12)
- Advise changes to physical environment to help make lifestyle change (e.g. create a more relaxing space in bedroom for better sleep) (15)
- Help patient with their self-belief in their ability to change (e.g. by visualizing themselves doing a wanted behaviour; or by using positive self-talk) (17)
- Help patient with their stress about a lifestyle change (e.g. anxiety about stopping smoking) by treating stress in the treatment session (20)
- Help patient with their stress about a lifestyle change (e.g. anxiety about stopping smoking) by teaching the patient stress-management tools to use by themselves (e.g. a breathing relaxation technique) (21)
- Advise change to social life to help make a lifestyle change (e.g. spend less time with heavy drinking colleagues) (22)
- Model positive lifestyle habits in your own life and share your experience with patients (24)

End of Block: Techniques and Intervention Ingredients

Start of Block: Demographics

Q35 Which of the following best describes your main style of acupuncture?

- Traditional Chinese Medicine (1)
- Eight Principles (2)
- Five Element (3)
- Japanese (4)
- Other, please describe here (5) ________________________________________________

| 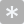 |
| --- |

Q36 What is your age?

________________________________________________________________

Q37 What is your gender

- Male (1)
- Female (2)
- Other (3)
- Prefer not to say (4)

Q38 How many years have you been in practice as an acupuncturist?

________________________________________________________________

Q39 In a typical week, approximately how many treatments do you give?

________________________________________________________________

Q40 In which of the ways listed below do you practise as an acupuncturist? Tick as many as apply

- Multidisciplinary clinic (1)
- Have own premises at home (2)
- Provide home visits (3)
- Rent a room (4)
- Acupuncture clinic (5)
- Multibed clinic (6)
- NHS (7)
- Volunteer (9)
- Other, please add details (8) ________________________________________________

End of Block: Demographics

Start of Block: Personal lifestyle habits

Q41 The following is an optional set of questions about your personal lifestyle habits. 


Please remember all of the data entered here is completely confidential and you do not need to provide any personal contact details if you choose not to.


If you prefer to miss any questions or the whole section please tick the 'prefer not to say' boxes or just skip to final page to claim your gift voucher.


Which of these apply to you?

- Smoker (1)
- Ex-smoker (2)
- Never smoked (3)
- Prefer not to say (4)

Q42 To answer the next 3 questions think about your eating habits during the past year.  Indicate how often you eat the following foods. Please include all meals, snacks, and food eaten out.


1.  Salad or vegetables

- Less than 1/week (1)
- 1/week (2)
- 2-3 times/week (3)
- 4-6 times/week (4)
- 1/day (5)
- 2 or more times/day (6)
- Prefer not to say (7)

|  |
| --- |

Q43 2. Fruit, including fresh, canned, or frozen, but not including juices

- Less than 1/week (1)
- 1/week (2)
- 2-3 times/week (3)
- 4-6 times/week (4)
- 1/day (5)
- 2 or more times/day (6)
- Prefer not to say (7)

|  |
| --- |

Q44 High-fibre cereals (such as muesli, porridge or branflakes) or other whole grains (such as wholewheat or rye bread, brown rice, wholewheat pasta)

- Less than 1/week (1)
- 1/week (2)
- 2-3 times/week (3)
- 4-6 times/week (4)
- 1/day (5)
- 2 or more times/day (6)
- Prefer not to say (7)

Q45
Thinking about the last year, in a typical week how many units of alcohol did you drink? 
  A pint of average strength beer, larger, stout, cider is 2.3 - 2.6 units A single measure of spirits (whiskey, vodka, gin, rum) is 1 unit A small glass (125ml) of 11% wine is 1.5 units A medium glass (175ml) of 13% wine is 2.3 units

- Number of units (1) ________________________________________________
- Prefer not to say (2)
- I never drink alcohol (3)

Q46 To answer the next 3 questions please indicate how many times per week you take part in the following activities for at least 30 minutes or more at a time.
 

1. Light exercise (minimal effort) such as light gardening and light housework (e.g. dusting, sweeping, hoovering) and leisurely walking (e.g. walking your dog).

- 0/week (1)
- 1-3 times/week (2)
- 4-7 times/week (3)
- 8 or more times/week (4)
- Prefer not to say (5)

|  |
| --- |

Q47
2. Moderate exercise (not exhausting), such as brisk walking, regular cycling, regular swimming, moderate gardening (e.g. raking, weeding, digging), dancing, tai-chi, yoga or moderate exercise classes.

- 0/week (1)
- 1-3 times/week (2)
- 4-7 times/week (3)
- 8 or more times/week (4)
- Prefer not to say (5)

|  |
| --- |

Q48 3. Vigorous exercise (heart beats rapidly), such as running, vigorous cycling, lap swimming, aerobics, heavy garden work, weight training, competitive sport (e.g. soccer, singles tennis)

- 0/week (1)
- 1-3 times/week (2)
- 4-7 times/week (3)
- 8 or more times/week (4)
- Prefer not to say (5)

End of Block: Personal lifestyle habits

Start of Block: Block 8

Q49 Thank you for taking the time to complete this survey. 

End of Block: Block 8
