# Supplementary material for: Lifestyle and health behaviour change support in traditional acupuncture: a mixed method survey study of reported practice (UK)
Source: BMC Complement Med Ther. 2022 Sep 21;22:248. doi: 10.1186/s12906-022-03719-6 (PMC9490899; doi:10.1186/s12906-022-03719-6)
Supplement: Supplementary file 2 — Additional file 2: Appendix 2. Development of TPB Scales [39, 40]. [file 12906_2022_3719_MOESM2_ESM.docx]

**Appendix 2: Development of TPB Scales**

Likert scale questions across four domains: Attitudes to Behaviour, Attitude to Clinical Role, Norms, and Perceived Behavioural Control (PBC) were analysed using a Principal Component Analysis (PCA) and Cronbach’s Reliability test. The PCA was conducted on the 18 items with oblique rotation (direct oblimin) to identify correlations. The Kaiser-Meyer-Olkin measure verified the sampling adequacy for the analysis (KMO = 0.769), and all KMO values for individual items were greater than 0.66 (above the acceptable limit of 0.5). ^39^

Factor 1 (Attitude to Clinical Role) was comprised of 5 items reported on a 7-point Likert scale that explained *29% of the variance* with factor loadings from .484 to .838. Scores ranged from 1-4.4 (Mean 1.80 SD 0.62). Factor 2 (Attitude to Behaviours) was comprised of 4 items reported on a 5-point Likert scale that explained *11.5% of the variance* with factor loadings from .604 to .786. Scores ranged from 1-5 (Mean 1.68 SD 0.56). Factor 3 (PBC) was comprised of 3 items reported on a 7-point Likert scale that explained *8.75% of the variance* with factor loadings from -.747 to -.890. Scores ranged from 1-7 (Mean 2.60 SD 1.01). Factor 4 (Norms) was comprised of 3 items reported on a 7-point Likert scale that explained *7% of the variance* with factor loadings from .660 to .879. Scores ranged from 1-5.33 (Mean 2.35 SD 0.88). The Cronbach’s α showed high reliabilities^40^ for these subscales: *Attitude to Clinical Role (*α = *.809)*; *Attitude to Behaviours (*α = .762); *Perceived Behavioural Control (*α = .762)*;* and *Norms (*α = .706)

5 factors had eigenvalues over Kaiser’s criterion of 1 and in combination explained 60.96% of the variance. The scree plot showed inflexions that would justify retaining both 3, 4 or and 5 factors, we retained 4 factors which were supported in previous TPB research, the 5^th^ factor was theoretically unsupported. One item (‘finding out about sleep hygiene behaviour’) clustered with 2 factors and was removed from the *Attitude to Behaviour* scale. The only two negatively-worded items (included in the *Attitude to Clinical Role* section) load together on a sixth factor and hence are excluded.

The Pattern Matrix Table shows the factor loadings after oblique rotation. The items that cluster on the same factor suggest that factor 1 represents *Attitude to Clinical Role (finding out about health behaviour)*, factor 2 represents *Attitude to Behaviour (importance of behaviour for health outcomes)*, factor 3 represents *PBC*, factor 4 represents *Norms (subjective norms regarding supporting behaviour change in acupuncture practice)* and factor 5 does not appear to clearly represent any construct.

| Pattern Matrix | | | | | | |
| --- | --- | --- | --- | --- | --- | --- |
|  |  | | | | | |
|  | 1 | 2 | 3 | 4 | 5 | 6 |
| Finding out about patients' smoking habits is a very important part of my work. | .838 |  |  |  |  |  |
| Finding out about patients' alcohol consumption is a very important part of my work. | .827 |  |  |  |  |  |
| Finding out about patients' sleep hygiene is a very important part of my work. | .608 | .369 |  |  |  |  |
| Finding out about patients' diet/eating habits is a very important part of my work. | .539 |  |  |  | -.368 |  |
| Finding out about patients' physical activity is a very important part of my work. | .484 |  |  |  |  |  |
| How important do you believe each of the following 5 lifestyle issues are for health? - Sleep |  | .786 |  |  |  |  |
| How important do you believe each of the following 5 lifestyle issues are for health? - PhysicalActivity |  | .749 |  |  |  |  |
| How important do you believe each of the following 5 lifestyle issues are for health? - Diet |  | .741 |  |  |  |  |
| How important do you believe each of the following 5 lifestyle issues are for health? - Alcohol |  | .604 |  |  | .528 |  |
| I feel properly trained to help patients make lifestyle changes |  |  | -.890 |  |  |  |
| I am confident in my ability to help patients change their lifestyle habits. |  |  | -.831 |  |  |  |
| I find it difficult to help patients make lifestyle changes |  |  | -.747 |  |  |  |
| My fellow acupuncturists would expect me to include lifestyle change in my treatments. |  |  |  | .879 |  |  |
| Most traditional acupuncturists include lifestyle change alongside their treatments |  |  |  | .806 |  |  |
| The acupuncture college I attended values working on lifestyle change within consultations. |  |  |  | .660 |  |  |
| How important do you believe each of the following 5 lifestyle issues are for health? - Smoking / Not smoking |  |  |  |  | .768 |  |
| I have no time to spend on lifestyle change with patients |  |  |  |  |  | -.801 |
| My work is to provide treatment, I leave lifestyle change to others. |  |  |  |  |  | -.725 |
| Extraction Method: Principal Component Analysis.  Rotation Method: Oblimin with Kaiser Normalization. | | | | | | |
| Imputation Number = 1 | | | | | | |
| Rotation converged in 18 iterations. | | | | | | |

| **Component Correlation Matrix** | | | | | |
| --- | --- | --- | --- | --- | --- |
| Component | 1 | 2 | 3 | 4 | 5 |
| 1 | 1.000 | .178 | -.345 | .274 | -.023 |
| 2 | .178 | 1.000 | -.180 | .115 | .151 |
| 3 | -.345 | -.180 | 1.000 | -.250 | -.023 |
| 4 | .274 | .115 | -.250 | 1.000 | -.017 |
| 5 | -.023 | .151 | -.023 | -.017 | 1.000 |
|  | | | | | |

Above shows that the principal components have a small relationships with each other (if the constructs were independent then oblique rotation should show same solution as orthogonal rotation and the components in above table would have correlation coefficients of 0), we cannot assume independence between components so obliquely rotated solution is more reasonable.
